# Experimental Study on Conductivity Anisotropy of Limestone Considering the Bedding Directional Effect in the Whole Process of Uniaxial Compression

**DOI:** 10.3390/ma9030165

**Published:** 2016-03-04

**Authors:** Xinji Xu, Bin Liu, Shucai Li, Lei Yang, Jie Song, Ming Li, Jie Mei

**Affiliations:** 1 Geotechnical and Structural Engineering Research Center, Shandong University, Jinan 250061, Shandong, China; xuxinji1990@163.com (X.X.); lishucai@sdu.edu.cn (S.L.); yanglei@sdu.edu.cn (L.Y.); tom1442@sina.com (J.S.); lim_zara@163.com (M.L.); meijie900220@163.com (J.M.); 2 School of Civil Engineering, Shandong University, Jinan 250061, Shandong, China

**Keywords:** bedding directional effect, uniaxial compression, potential difference, anisotropy, regression analysis

## Abstract

Experimental studies were conducted on the changes of the potential differences in different directions during the uniaxial compression on limestone samples parallel and normal to the bedding plane. In the test, electric current was supplied at both ends of the samples, and concurrent measurement was conducted in four measuring lines at a 45-degree angle to each other. First, the change laws of the potential differences in different directions and the similarities and differences of rock samples were summarized. In regards to the uniaxial compression properties and crack growth, the above-mentioned similarities and differences were further analyzed. Then, the anisotropy factor was introduced to further explore the response characteristics. It was found that the anisotropic changes of rock samples went through three stages during the uniaxial compression process, providing a reference for describing the properties in different failure stages of rock samples and obtaining precursory information about the fracture. Besides, the relationship between the peak stress and initial potential difference in a direction normal to the current direction was obtained by means of data fitting, providing a new method of predicting the uniaxial compressive strength of rock samples. According to the preceding analysis, this paper studied rock anisotropy by considering the bedding directional effect in terms of conductivity and provided a reference for subsequent study on rock materials’ properties and engineering practices.

## 1. Introduction

Rock is a common material in civil engineering and geotechnical engineering. It is well known that rock usually shows obvious anisotropic characteristics due to its very complex composition and internal structure. The anisotropy is reflected not only in its mechanical properties [1,2,3] like the strength and deformation but also in its geophysical properties like the wave velocity and conductivity. Many scholars have made much effort in this regard and accomplished a great deal. As for studies on the anisotropy of the rock conductivity, scholars have mainly examined the anisotropic characteristics of the conductivity during the failure process by means of numerical simulations [4,5], laboratory tests and field measurements [6,7]. Specifically, Zhu *et al.* [8] and Wang *et al.* [9] summarized the existing studies in terms of the preceding aspects and reviewed their application in studies on earthquake monitoring and prediction.

As early as 1968, Brace and Orange [10] measured the changes of the horizontal and normal electrical resistivity during the loading process of rock samples by employing the two-electrode method but did not perform further studies. The two-electrode method measures the average electrical resistivity between two end faces of rock samples, which can hardly reflect the directivity of changes of the electrical resistivity and is not applicable to laboratory tests and studies on the anisotropy of the electrical resistivity. Therefore, Zhang and Lu [11] employed the symmetric four-electrode method. Specifically, three measuring lines were laid in parallel with the maximum principal stress and intermediate principal stress, as well as at a 45-degree angle with the maximum principal stress to study the changes of the electrical resistivity of saturated sandstone in different triaxial stress and strain rate conditions but did not further study the anisotropy of the changes. Employing the same measuring method, Chen *et al.* [12] selected cuboid water-saturated rock samples without external water recharge to study the relationship between the anisotropy of the apparent resistivity and pressure. They found that the electrical resistivity was closely related to the rupture direction of rock and deduced a formula that determined the main rupture direction of rock by using a principal axis of the anisotropy of the changes of the electrical resistivity. Further considering the action of recharging water, An *et al.* [13] employed the symmetric four-electrode method and four measuring lines forming a 45-degree angle with each other to measure the electrical resistivity of cuboid granite samples of different sizes during the uniaxial compression process. They found that the changes of the electrical resistivity showed obvious directivity. Moreover, the direction of the anisotropy principal axis would jump regularly as the pressure increased. According to the study results of Chen [14], the electrical resistivity of rock showed the smallest changes in the pressure direction, greatest changes in a direction normal to the pressure, and intermediate changes at a 45-degree angle with the pressure direction.

Based on the studies of Chen *et al.* [12], Chen *et al.* [15,16,17,18] gradually developed and improved the method of determining the main rupture direction of rock by using the anisotropy of the changes of the rock electrical resistivity. They performed a series of experimental studies on the anisotropy of the electrical resistivity of rock samples in uniaxial, low-confining-pressure triaxial and shear conditions by means of combined measurement of the anisotropy of the apparent resistivity, electrical profiling and electrical sounding. The results indicated that the direction of the anisotropy principal axis with the largest changes of the apparent resistivity of rock was consistent with the main rupture direction of rock. Moreover, it was found that the apparent resistivity showed obvious anisotropic changes when the crack and fracture zone passed through the measuring field. The directions of the four anisotropy principal axes obtained by using four combinations tended to be consistent and agreed with the direction of the fracture zone. When the crack and fracture zone did not pass through the measuring field, the directions of the four anisotropy principal axes were inconsistent, or no anisotropy solution could be obtained. The latter was shown most obviously, when the crack plane was parallel to the measuring plane.

Above all, most of the existing methods of measuring the anisotropy of the conductivity adopt the symmetrical four-electrode arrangement mode. That is, centering on the central point of the measuring plane of rock samples, four measuring lines are systematically laid out at a 45-degree angle with each other. However, the electric current supply is located on the surface of rock samples in this method. As a result, the distribution of the electric field in rock samples is uneven, and the current route concentrates near the measuring plane instead of traversing the middle zone of rock samples. Therefore, the measurement results are greatly affected by the conductivity of local areas near the selected measuring plane, which can hardly reflect the overall changes of the conductivity of rock samples. Moreover, the measurement in the four measuring lines can only be carried out respectively to avoid the mutual interference between different measuring lines. The electric current is supplied to, and the measurement is conducted in only one measuring line each time, and concurrent collection in multiple measuring lines cannot be realized, which to some extent restricts the number of sampling points. In addition, most of the preceding conductivity anisotropic tests did not consider the impact of the bedding directional effect of rock. The existing study results indicated that the mechanical properties of rock had an obvious bedding directional effect [1,2,3]. In regards to geophysical properties, a series of studies were conducted on the velocities of the compression wave and shear wave in different bedding directions [19,20,21,22]. In contrast, studies on the anisotropy of rock conductivity by taking the bedding directional effect into consideration have not been reported.

Therefore, this paper studied the changes of the potential differences in different directions during the whole process of uniaxial compression on limestone samples parallel and normal to the bedding plane. In this test, electric current was supplied at both ends of the samples, and concurrent measurement were made in four measuring lines at a 45-degree angle with each other. First, the change laws of the potential differences in different directions, and the similarities and differences of rock samples parallel and normal to the bedding plane were summarized. From the view of uniaxial compression properties and crack growth, the above-mentioned similarities and differences were further analyzed. Then, the anisotropy factor was introduced to explore the response characteristics of the potential difference in each direction to the failure process of the samples, and find their common laws. Finally, the relationship between the peak stress and initial potential difference in a direction normal to the current direction was obtained by means of data fitting. This paper aimed to introduce some new ideas to experimental studies on the anisotropy of rock by considering the bedding directional effect in terms of conductivity, and provide a reference for subsequent study on rock materials’ properties and engineering practices.

## 2. Test Device and Method

In this paper, limestone samples from a tunnel in Guangxi, China were selected as the test material. The samples were cored in a direction parallel or normal to the bedding direction and then processed into standard cylinders with a size of Φ 50 mm × 100 mm. The sample surface is smooth and preparations meet related code requirements [23].

**Figure 1:**
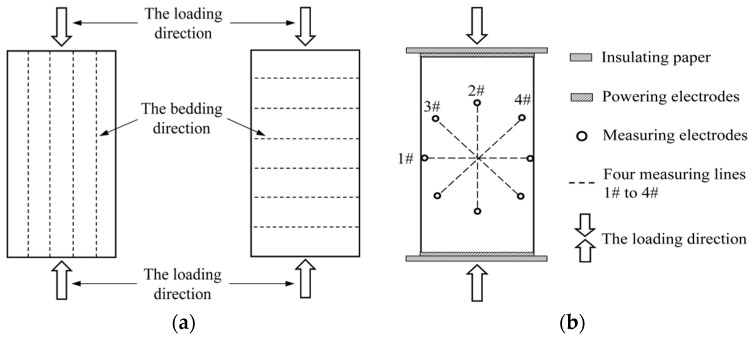
Schematic diagram of test device and method. (**a**) Relationship between the loading and bedding direction; (**b**) Measuring method.

The relationship between the loading direction and bedding direction is shown in Figure 1a. The left one indicates rock samples parallel to the bedding direction and the right one indicates rock samples normal to the bedding direction. The test was arranged as follows: as shown in Figure 1b, cooper electrodes were fixed at both ends of rock samples as the powering electrode; the insulating paper was used to prevent current spreading along the rock testing machine; for the measuring electrode, centering on the central point of the rock sample surface, four measuring lines 1#–4# were symmetrically laid out at a 45-degree angle to each other. Measuring electrodes were directly glued in appropriate positions on the rock sample surface with the silver conductive adhesive, about 5 cm apart from each other. To reduce the contact resistance, the powering and measuring electrodes should be coupled with rock samples by using clay. In the measurement, a hypervelocity real-time collection device stated in [24] was used. A 32 V constant voltage was applied to both ends of the powering electrode via the dry battery box, and each channel of the hypervelocity real-time collection device collected the potential differences between measuring electrodes in different directions simultaneously. The changes of the potential difference during the loading process could reflect the growth of cracks inside rock samples and the resulting changes of the rock internal structure.

**Figure 2:**
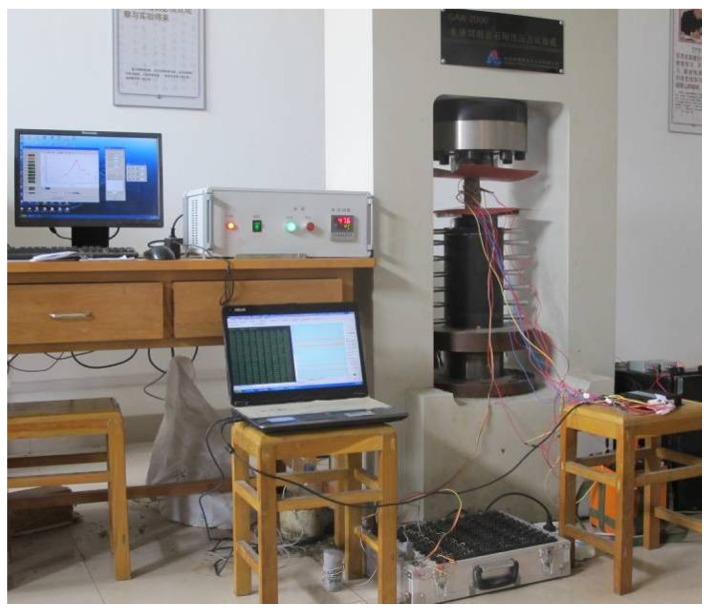
The test system.

A total of 10 rock samples were divided into two groups with five in each group, one group with the rock axis parallel to the bedding direction while the other with the rock axis normal to the bedding direction. The uniaxial compression test was conducted on the two groups of rock samples one by one. In the uniaxial compression test, the electro-hydraulic servo rock rigid testing machine was used for loading, and the axial displacement was selected to control the loading. After trial and error, the loading rate was controlled to 0.2 mm/min until the rock sample finally failed. Figure 2 shows the picture of the testing system.

## 3. Test Results

**Figure 3:**
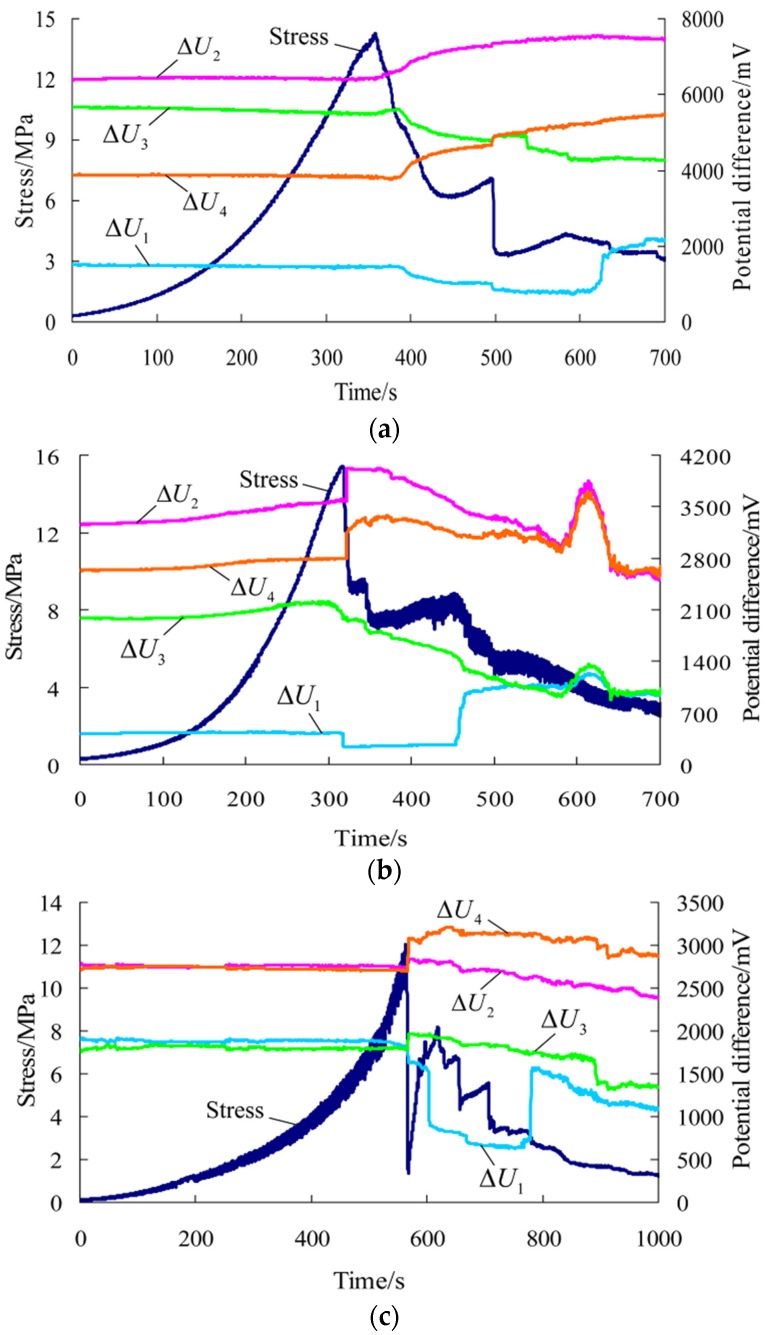
Stress-time curve and time-varying curve of the potential difference in each direction during the whole process of uniaxial compression for rock samples parallel to the bedding direction. (**a**) Rock sample 6-1; (**b**) Rock sample 7-1; (**c**) Rock sample 7-3.

**Figure 4:**
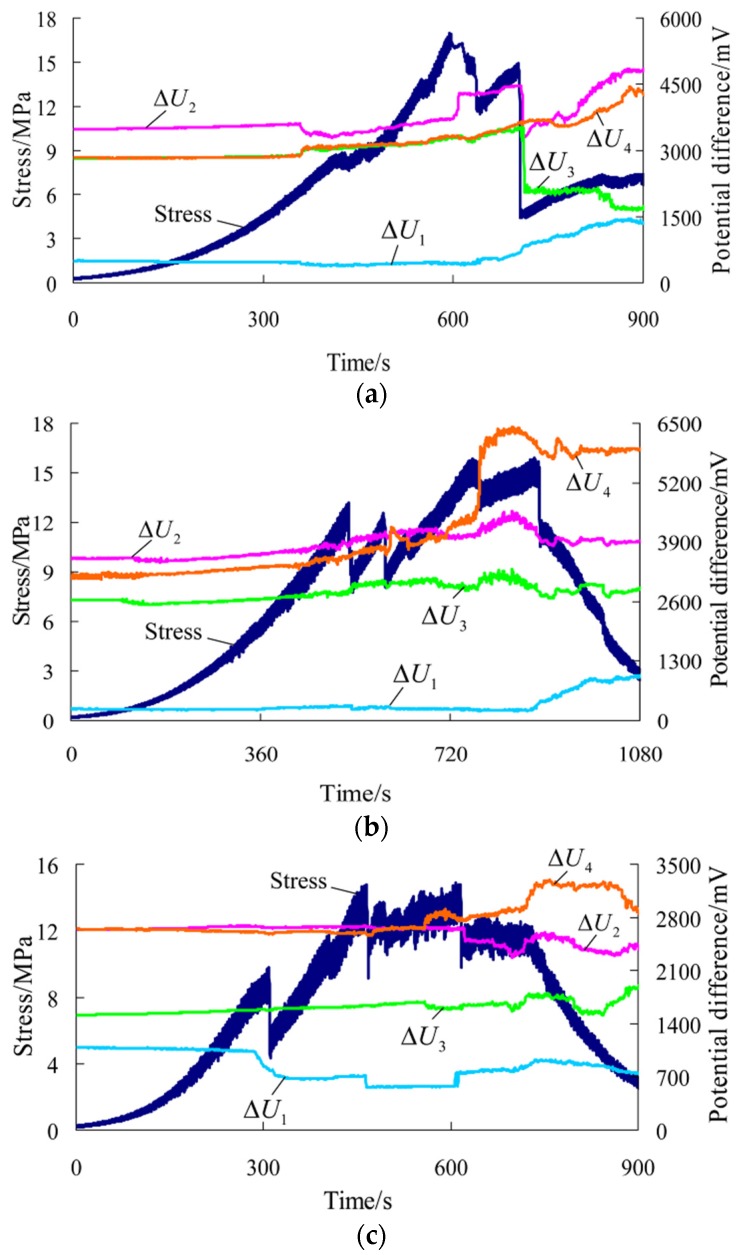
Stress-time curve and time-varying curve of the potential difference in each direction during the whole process of uniaxial compression for rock samples normal to the bedding direction. (**a**) Rock sample 5-1; (**b**) Rock sample 5-2; (**c**) Rock sample 6-4.

**Figure 5:**
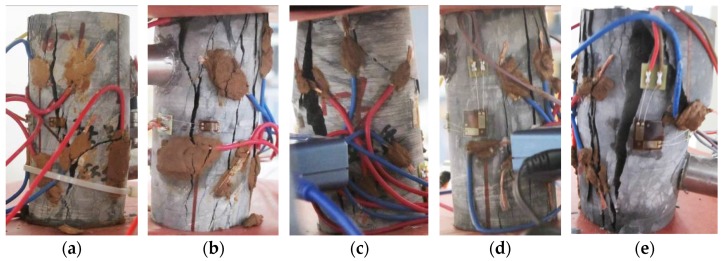
Failure state of rock samples parallel to the bedding direction. (**a**) Rock sample 6-1; (**b**) Rock sample 6-5; (**c**) Rock sample 7-1; (**d**) Rock sample 7-2; (**e**) Rock sample 7-3.

**Figure 6:**
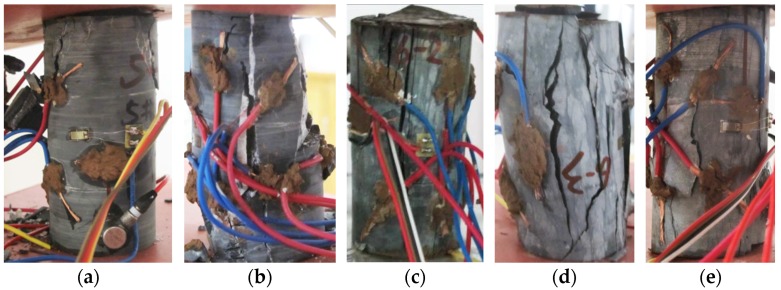
Failure state of rock samples normal to the bedding direction. (**a**) Rock sample 5-1; (**b**) Rock sample 5-2; (**c**) Rock sample 6-2; (**d**) Rock sample 6-3; (**e**) Rock sample 6-4.

After tests, the results and related parameters of all rock samples were listed in Table 1 and Table 2, respectively. The coefficient of variation in the tables is the ratio of the standard deviation to the mean of a group of data, which can reflect the degree of dispersion of data. According to the test results and sampling, six representative rock samples (three parallel to and three normal to the bedding) were selected to draw the stress-time curve and time-varying curve of the potential difference in each direction during the whole process of uniaxial compression, as shown in Figure 3 and Figure 4 (the other four rock samples are shown in Figures S1 and S2). $\Delta U_{1}$−$\Delta U_{4}$ correspond to the changes of the potential differences measured in measuring lines 1#–4# respectively. Figure 5 and Figure 6 show the pictures of the failure state of the two groups of rock samples.

## 4. Test Result Analysis

### 4.1. Analysis on the Conductivity of Rock Samples in Uniaxial Compression

(1) After comparative analysis on the changes of the potential difference in each direction during the whole process of uniaxial compression on limestone samples, and according to test data of other rock samples, the following laws were found:

For rock samples parallel to the bedding, the peak strain ranges from 0.98% to 1.841%, with an average of 1.327%. This kind of failure belongs to brittle failure. In the stress-time curve, there are five obvious stages: compaction, elastic, plastic, fracture and residual strain. Moreover, the curve shows only one peak; the pre-peak curve is smooth and indicates stable mechanical properties; the post-peak curve shows multi-level falls and indicates slowly decreasing bearing capacity.

In the corresponding uniaxial compression process, the potential difference in each direction of rock samples parallel to the bedding changes is as follows: (a) Prior to the stress peak, the potential difference in each direction basically remains unchanged or rises slightly; (b) When the stress reaches the peak and macro fracture occurs, the potential differences in measuring lines 2# and 4# rise suddenly; that in measuring line 1# declines suddenly; that in measuring line 3# rises in some rock samples while declines in others; (c) In the post-peak stage, the potential differences in different directions change differently, that is, constant rise, constant decline or fluctuation. As for the relationship with the fracture of rock samples, the potential difference in a direction changes abruptly when the stress declines while that in another direction does not respond obviously to the decline in stress. In general, the post-peak curve shows different forms.

For rock samples normal to the bedding, the peak strain ranges from 1.466% to 2.881%, with an average of 2.013%, which is about 1.5 times of that of rock samples parallel to the bedding. This is caused by the accumulated deformation of each bedding plane. According to the average peak strain, we find that the rock samples parallel to the bedding are closer to brittle failure, while the rock samples normal to the bedding are closer to ductile failure. So, the stress behavior *versus* time is very different in the two orientations. For rock samples normal to the bedding, at the end of the elastic stage or at the beginning of the plastic stage, local fracture occurs multiple times, and the uniaxial compression curve shows obvious multiple peaks. Moreover, the post-peak bearing capacity declines rapidly.

The potential difference in each direction of rock samples normal to the bedding changes as follows: (a) Prior to the first macro fracture, the potential difference in each direction basically remains unchanged or starts to rise gradually; (b) After that, local fracture occurs on rock samples multiple times, and the stress-time curve shows multiple peaks. During this stage, the potential differences in measuring lines 2# and 4# are on the rise as a whole while those in measuring lines 1# and 3# rise in some rock samples and decline in others. Moreover, the potential difference in each direction has different levels of sensitivity to the moment of each fracture. Some rise or decline suddenly while others do not show obvious changes at the moment of a drop in stress. However, in general, the potential difference is responsive to the last fracture of rock samples in varying degrees; (c) In the post-peak stage, the potential difference in each direction changes differently and the curve shows varied forms.

(2) According to the preceding analysis, for rock samples parallel and normal to the bedding, the changes of the potential difference in each direction in uniaxial compression have the following in common: (a) Prior to the first macro fracture, the potential difference in each direction basically remains unchanged or starts to rise gradually; (b) At the moment of failure due to stress peak of rock samples parallel to the bedding and during multiple local fractures, the potential differences in measuring lines 2# and 4# show a rise; (c) In the post-peak stage, the potential difference in each direction changes differently and the curve shows varied forms.

On the other hand, for the two groups of rock samples, the changes of the potential difference in each direction in uniaxial compression have the following differences: (a) At the moment of failure due to stress peak of rock samples parallel to the bedding and during multiple local fractures, the potential differences in measuring lines 1# and 3# change differently; (b) For rock samples parallel to the bedding, the potential difference in each direction shows obvious sudden changes as failure occurs at the peak. For rock samples normal to the bedding, the potential difference in each direction has different levels of sensitivity to the moment of each fracture during multiple local fractures but reacts obviously to the last fracture.

The changes of the conductivity during the loading process of rock samples are closely related to rock deformation and failure conditions. The compressive deformation of rock samples, crack initiation, propagation and transfixion change the internal structure of rock samples as well as the connectivity of the conductor and the degree of contact between rock minerals, thus greatly affecting the overall conductivity of rock samples [25]. Therefore, starting with the perspective of uniaxial compression properties of rock samples and crack growth can help to gain a clearer understanding of the differences and similarities of the changes of the preceding potential differences. According to the test results, the failure process of uniaxial compression of rock samples can be broadly divided into three stages:

(a) Prior to the first macro fracture, rock samples go through the compaction and elastic deformation stage. For most rock samples, no new cracks initiate in this stage [26,27,28,29], and rock samples hold their original conductivity. Therefore, for rock samples parallel and normal to the bedding, the potential difference in each direction remains unchanged, and for a small number of rock samples, micro cracks initiate locally in the elastic stage, resulting in the gradual rise of the potential difference.

(b) From the plastic stage, new cracks inside rock samples constantly initiate and stably propagate. Then, cracks gradually interconnect and unite to form macro cracks and cause macro fracture to rock samples [26,27,28,29,30,31]. For rock samples parallel to the bedding, the stress has an obvious sudden fall at the peak, and then the uniaxial compression curve shows multi-level falls with the constant occurrence of small-scale fracture events. For rock samples normal to the bedding, obvious local fracture occurs multiple times, and the uniaxial compression curve shows multiple peaks.

In general, the changes of cracks inside rock samples are relatively complex in this stage. In particular, at the moment of the occurrence of macro fracture, the internal structure of rock samples changes suddenly, and the conductivity of rock samples changes accordingly. However, for rock samples parallel and normal to the bedding, the potential difference in each direction changes differently and has different levels of sensitivity to the moment of fracture of rock samples. According to the test process, it is believed that the preceding differences are related to whether the macro fracture plane passes through the measuring field. When the fracture plane passes through a measuring line, the potential difference in this line will suddenly change while the changes of the potential difference are not obvious in the other three measuring lines without fracture plane passing through. In particular, when the fracture plane does not pass through the measuring field totally or is parallel to the measuring plane, the changes of the potential difference in each direction are relatively slight and are not sensitive to the fracture.

(c) In the post-peak stage, the bearing capacity of rock samples shows multi-level falls and finally tends to be stable. In this stage, for rock samples parallel and normal to the bedding, the conductivity and the potential difference in each direction change differently, and the curve shows varied forms.

### 4.2. Analysis of the Anisotropic Change Laws of the Conductivity of Rock Samples in Uniaxial Compression

In uniaxial compression of rock samples, more concern is given to the macro fracture stage of rock samples. In this stage, for rock samples parallel and normal to the bedding, the changes of the potential difference in each direction have more differences than similarities. To further explore the response characteristics of the potential difference in each direction to the fracture of rock samples, this paper refers to the analysis method in [19,22], and introduces the anisotropy factor λ, which is defined as the ratio of the potential difference in another direction (namely, one of the directions of measuring lines 2#–4#) to that normal to the current direction (namely, direction of measuring line 1#), that is:

(1) $$\lambda_{2}=\frac{\Delta U_{2}}{\Delta U_{1}},\;\lambda_{3}=\frac{\Delta U_{3}}{\Delta U_{1}},\;\lambda_{4}=\frac{\Delta U_{4}}{\Delta U_{1}}$$

**Figure 7:**
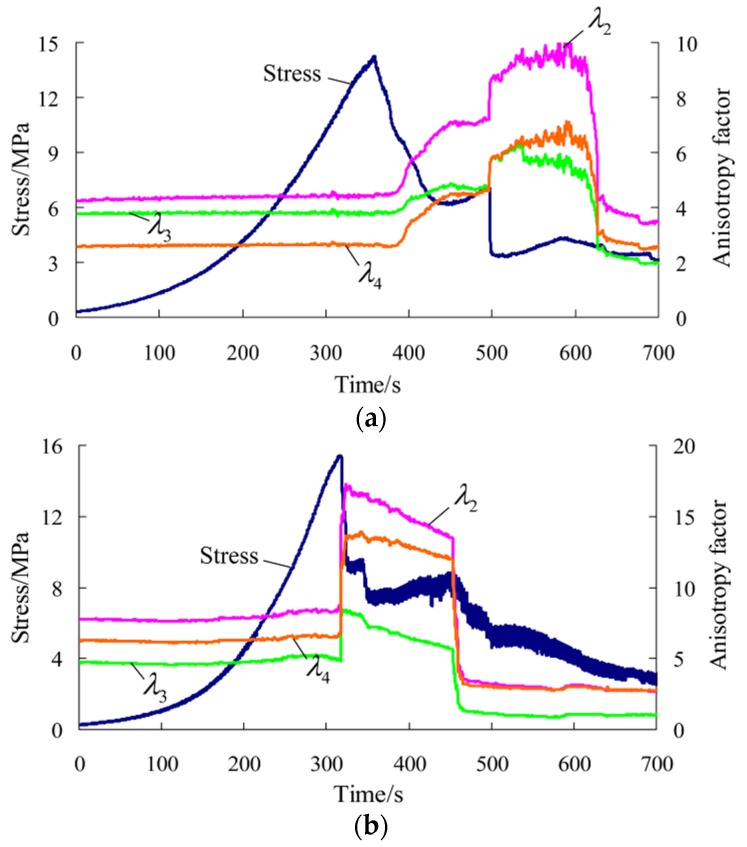

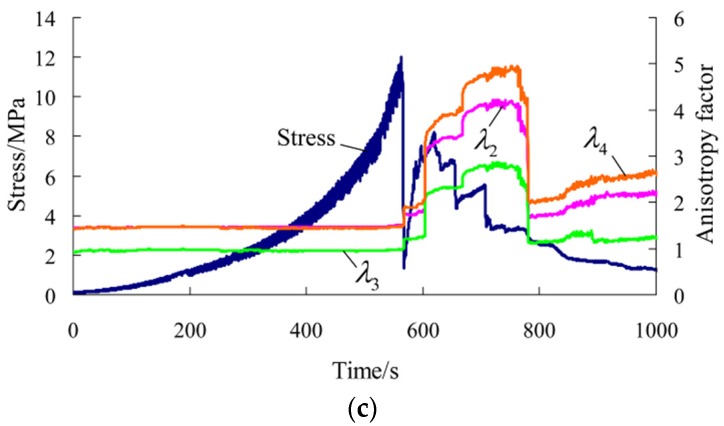
Stress-time curve and time-varying curve of the anisotropy factor in each direction during the whole process of uniaxial compression for rock samples parallel to the bedding direction. (**a**) Rock sample 6-1; (**b**) Rock sample 7-1; (**c**) Rock sample 7-3.

**Figure 8:**
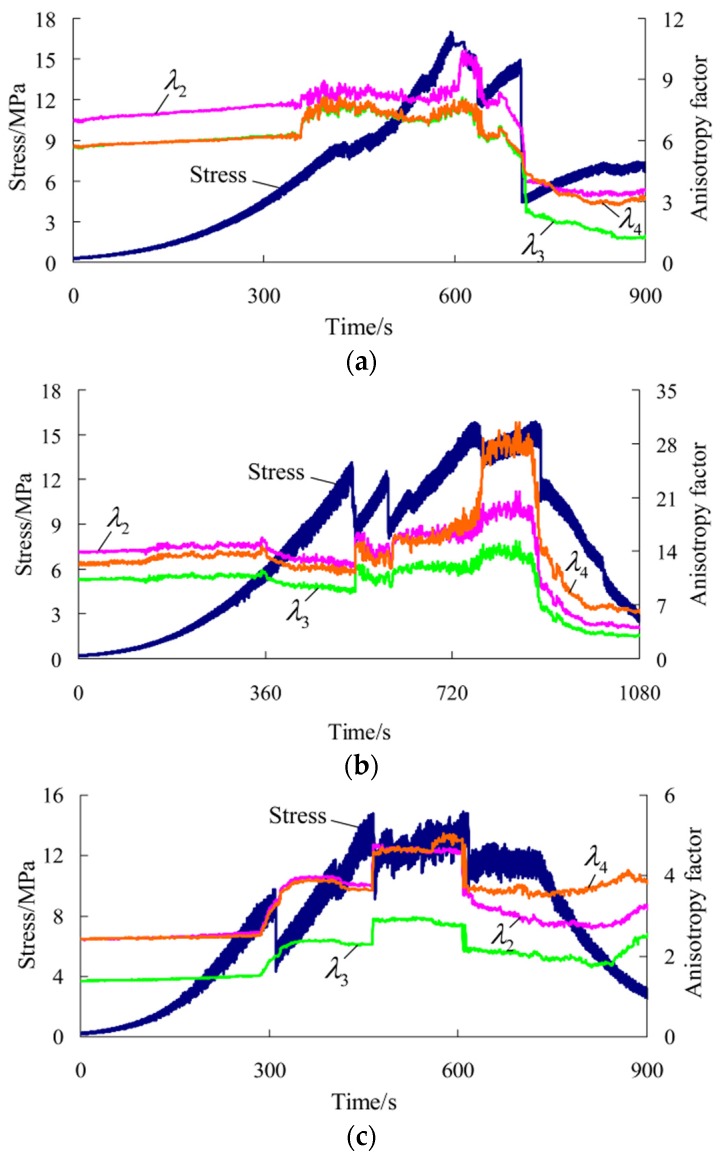
Stress-time curve and time-varying curve of the anisotropy factor in each direction during the whole process of uniaxial compression for rock samples normal to the bedding direction. (**a**) Rock sample 5-1; (**b**) Rock sample 5-2; (**c**) Rock sample 6-4.

Thus, the time-varying curve of the anisotropy factor in each direction in uniaxial compression of rock samples, and its corresponding relationship with the stress-time curve can be drawn, as shown in Figure 7 and Figure 8.

According to the preceding figures, the following can be obtained: (a) Prior to the macro fracture of rock samples, the anisotropy factor in each direction basically remains unchanged, which is nearly the same as the change laws of the potential difference described previously; (b) During the macro fracture process, the anisotropy factor in each direction shows obvious sudden changes. Moreover, it is very sensitive to the moment of failure at the peak and that of stress fall after the peak of rock samples parallel to the bedding, as well as the moment of multiple local fractures of rock samples normal to the bedding. In particular, the anisotropy factor rises obviously at the moment of the first macro fracture of rock samples and declines obviously at the moment of the last macro fracture; (c) In the post-peak stage, the anisotropy factor changes smoothly without quite obvious fluctuations.

It can be seen that the common laws during the changes of the potential difference in each direction of rock samples parallel and normal to the bedding are found by introducing the anisotropy factor. It is found that the anisotropic changes of rock samples go through three stages in uniaxial compression. The anisotropy remains unchanged prior to the macro fracture and shows sudden changes at the moment of the macro fracture. In particular, the anisotropy rises at the moment of the first macro fracture, declines at the moment of the last macro fracture, and tends to be stable in the post-peak stage. The anisotropy of the conductivity provides a reference for describing the properties in different failure stages of rock samples and obtaining the precursory information about the fracture instability.

### 4.3. Relationship between the Initial Potential Difference Normal to the Current Direction and the Uniaxial Compressive Strength

According to Table 1 and Table 2, regardless of rock samples parallel or normal to the bedding, measuring line 1# has the initial potential difference before loading starts; moreover, the initial potential differences of different rock samples are significantly varied and show obvious discrete properties. If a homogeneous isotropic material is measured by the method in this test, the initial potential difference in the direction of measuring line 1# shall be 0. However, in this test, this value is not 0. It can be inferred that the existence of the initial potential difference of measuring line 1# is caused by the inhomogeneity of rock materials. This is because different degrees of initial cracks exist inside rock samples, and the existence will inevitably affect the uniaxial compressive strength of rock samples.

**Figure 9:**
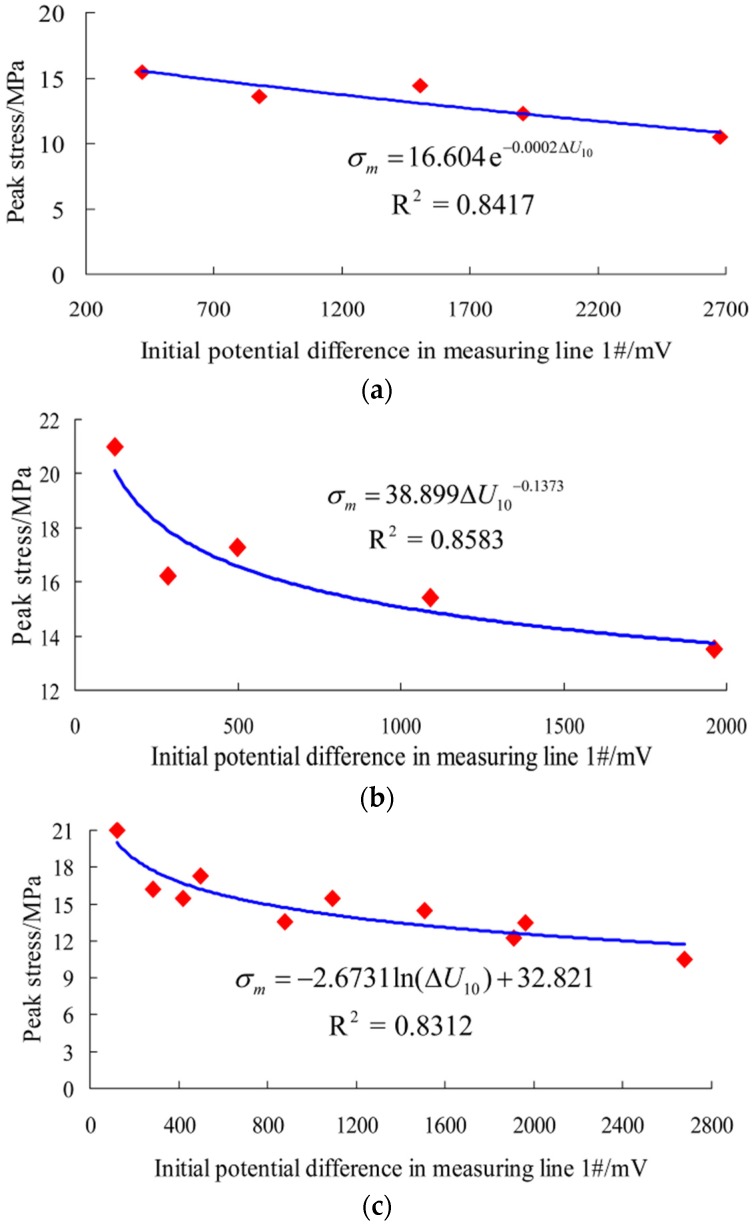
Relation curves between the peak stress and the initial potential difference in measuring line 1#. (**a**) Rock samples parallel to the bedding direction; (**b**) Rock samples normal to the bedding direction; (**c**) All samples.

In this test, the relationship between the peak stress of rock samples and the initial potential difference in measuring line 1# is obtained by fitting data using functions like linear, logarithm, power and exponent, respectively. After comparison of correlation coefficients (see Table 3), it is found that there is a strong correlation between the peak stress of rock samples and the initial potential difference of measuring line 1# for rock samples parallel and normal to the bedding. The function form with the largest correlation coefficient is selected as the regression equation to describe the relationship between them. Specifically, $\sigma_{m}=16.604e^{-0.0002\Delta U_{10}}$ is used as the regression equation of rock samples parallel to the bedding; $\sigma_{m}=38.899\Delta {U_{10}}^{-0.1373}$ is used as that of rock samples normal to the bedding; $\sigma_{m}=-2.6731\ln(\Delta U_{10})+32.821$ is used as that of all rock samples. They are drawn respectively in Figure 9.

According to Figure 9, although the curve shape is different in rock samples parallel and normal to the bedding direction, its change trend is the same. As the initial potential difference of measuring line 1# increases, the peak stress of rock samples decreases accordingly and finally tends to be stable. This is because the size of the potential difference of measuring line 1# is directly proportional to the degree of growth of the initial cracks inside rock samples. That is, the larger the initial potential difference of measuring line 1# and the higher degree of initial crack growth, the lower the resulting peak stress. This relationship can be used to predict the uniaxial compressive strength of rock samples, and the result can provide a reference for mechanical tests and necessary information for selecting samples and determining the loading size and multi-stage loading [32]. However, detailed studies are still required to get a more accurate prediction result.

## 5. Discussion and Conclusions

### 5.1. Discussion

From this paper, we can see that the changes of the potential difference in each direction are closely related to rock failure conditions and the bedding directions. In future, further research should be continued. Using the test method in this paper, more comprehensive and systematic studies could be conducted on other kinds of rock. Further studies on the impact of the bedding directional effect on the anisotropy of the conductivity by considering the intersection of the loading and bedding directions with other perspectives will be performed. In addition to laboratory tests, field experimental study should also be carried out to make this testing method more practical. During the construction process in geotechnical engineering, we can conduct long-term monitoring of the rock conductivity in different directions. Combined with the anisotropy characteristics obtained in laboratory tests, we can use the rock conductivity in different directions to reflect the rock damage state and degree of stability. It is an effective, non-destructive testing method, which helps to ensure construction safety.

### 5.2. Conclusions

Based on previous studies, this paper conducted experimental studies on the anisotropy of the conductivity of limestone samples parallel and normal to the bedding during the entire uniaxial compression process, and reached the following conclusions:

(1) The changes of the conductivity during the loading process of rock samples were closely related to rock deformation and failure conditions. The compressive deformation of rock samples, crack initiation, propagation and transfixion changed the internal structure of rock samples as well as the connectivity of the conductor and the degree of contact between rock minerals, thus greatly affecting the conductivity of rock samples. Due to the impact of the bedding directional effect, the changes of the potential difference in each direction of rock samples parallel and normal to the bedding had both similarities and differences in uniaxial compression.

(2) From the view of uniaxial compression properties and crack growth, the above-mentioned similarities and differences were further summarized. Prior to the first obvious fracture of rock samples, cracks grew stably, and the potential difference in each direction of rock samples parallel and normal to the bedding basically remained unchanged or gradually rose latterly. During the failure process of rock samples, new cracks inside rock samples were constantly initiated and stably propagated. Then, cracks gradually interconnected and united to form macro cracks and caused macro fracturing to rock samples. The correlation between the macro fracture plane and the measuring field resulted in different changes of the potential difference in each direction of rock samples parallel and normal to the bedding and varied levels of sensitivity to the moment of fracture of rock samples. In the post-peak stage, the bearing capacity of rock samples showed multi-level falls and finally tended to be stable. In this stage, for rock samples parallel and normal to the bedding, the conductivity and the potential difference in each direction changed differently, and the curve showed varied forms.

(3) The anisotropy factor was introduced to explore the response characteristics of the potential difference in each direction to the failure process of the samples, and find their common laws. It was found that the anisotropic changes of rock samples went through three stages during the uniaxial compression process. The anisotropy remained unchanged prior to the macro fracture and showed sudden changes at the moment of the macro fracture. In particular, the anisotropy rose at the moment of the first macro fracture, declined at the moment of the last macro fracture, and tended to be stable in the post-peak stage. The anisotropy of the conductivity provides a reference for describing the properties in different failure stages of rock samples and obtaining precursory information about fracture instability.

(4) The relationship between the peak stress and initial potential difference normal to the current direction was obtained by means of data fitting. This indicated that the size of the potential difference normal to the current direction was directly proportional to the degree of growth of the initial cracks. That is, the larger the initial potential difference and the higher degree of initial crack growth, the lower the resulting peak stress.

## Figures and Tables

**Table 1:** Test results of rock samples parallel to the bedding direction in uniaxial compression test.

Serial Number	Diameter (*D*/mm)	Height (*H*/mm)	Peak Stress (σ_m_/MPa)	Peak Strain (ε_m_/10^−3^)	Initial Potential Difference in Measuring Line 1# (Δ*U*_10_/mV)
6-1	49.10	103.00	14.43	11.46	1506.21
6-5	49.18	103.11	10.46	16.25	2677.00
7-1	49.16	101.82	15.47	10.45	419.80
7-2	49.22	102.90	13.60	9.80	877.00
7-3	49.00	102.58	12.26	18.41	1907.95
Average	49.13	102.68	13.24	13.27	1477.59
Variation coefficient	0.08	0.47	1.74	3.42	787.94

**Table 2:** Test results of rock samples normal to the bedding direction in uniaxial compression test.

Serial Number	Diameter (*D*/mm)	Height (*H*/mm)	Peak Stress (σ_m_/MPa)	Peak Strain (ε_m_/10^−3^)	Initial Potential Difference in Measuring Line 1# (Δ*U*_10_/mV)
5-1	49.28	101.70	17.26	19.75	499.45
5-2	49.04	101.58	16.20	28.81	285.43
6-2	49.16	102.50	20.98	14.66	121.83
6-3	49.21	102.22	13.50	17.34	1960.92
6-4	49.00	102.34	15.42	20.09	1090.60
Average	49.14	102.07	16.67	20.13	791.65
Variation coefficient	0.10	0.36	2.48	4.76	670.35

**Table 3:** The correlation coefficients of different regression equations for rock samples.

Regression Equation Type	Rock Samples Parallel to the Bedding Direction	Rock Samples Normal to the Bedding Direction	All Samples
Linear	0.8400	0.6893	0.7400
Logarithm	0.7397	0.8430	0.8312
Power	0.7175	0.8583	0.8089
Exponent	0.8417	0.7470	0.7939

## Supplementary Materials

The following are available online at www.mdpi.com/1996-1944/9/3/165/s1. Figure S1: Stress-time curve and time-varying curve of the potential difference in each direction during the whole process of uniaxial compression for rock samples parallel to the bedding direction. (a) Rock sample 6-5; (b) Rock sample 7-2, Figure S2: Stress-time curve and time-varying curve of the potential difference in each direction during the whole process of uniaxial compression for rock samples normal to the bedding direction. (a) Rock sample 6-2; (b) Rock sample 6-3.
