# Supplementary material for: Experimental Study on Conductivity Anisotropy of Limestone Considering the Bedding Directional Effect in the Whole Process of Uniaxial Compression
Source: Materials (Basel). 2016 Mar 4;9(3):165. doi: 10.3390/ma9030165 (PMC5456710; doi:10.3390/ma9030165)
Supplement: Supplementary file 1 [file materials-09-00165-s001.pdf]

# Supplementary Materials: Experimental Study on Conductivity Anisotropy of Limestone Considering the Bedding Directional Effect in the Whole Process of Uniaxial Compression

**Xinji Xu, Bin Liu, Shucai Li, Lei Yang, Jie Song, Ming Li and Jie Mei**

To control the article length, we just use six representative rock samples in this paper. The other four figures are shown as following.

Two rock samples parallel to the bedding direction:

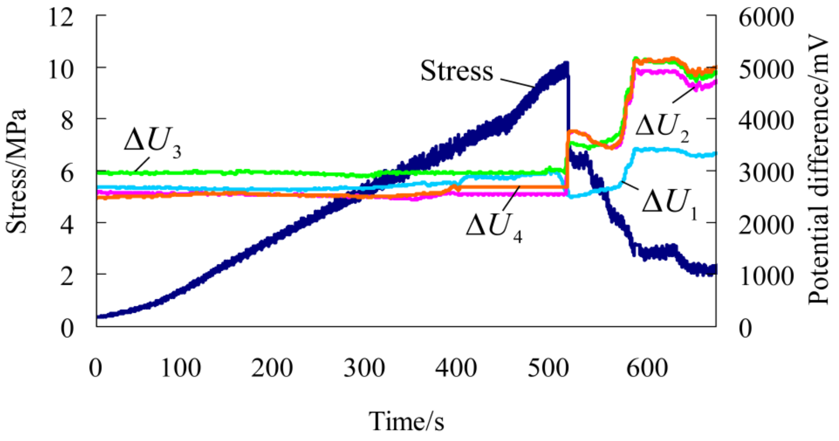


(**a**)

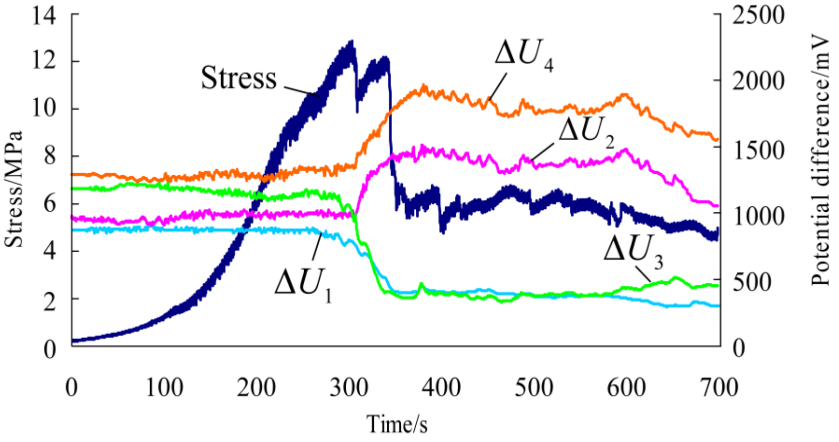


(**b**)

**Figure S1.** Stress-time curve and time-varying curve of the potential difference in each direction during the whole process of uniaxial compression for rock samples parallel to the bedding direction. (**a**) Rock sample 6-5; (**b**) Rock sample 7-2.

The other two rock samples normal to the bedding direction:

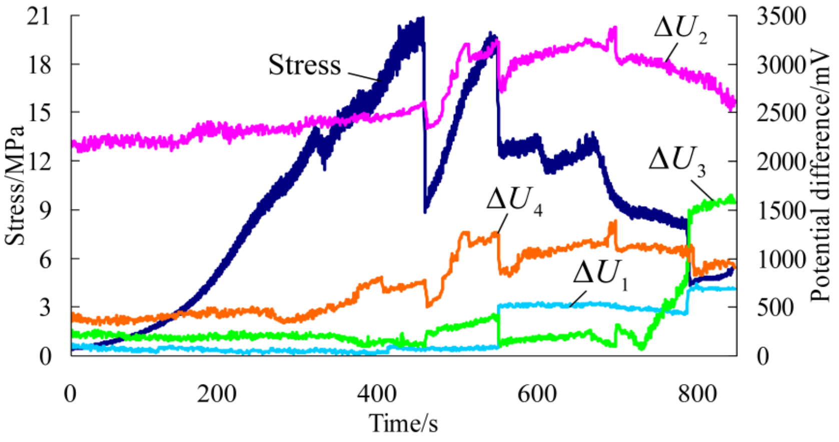


(**a**)

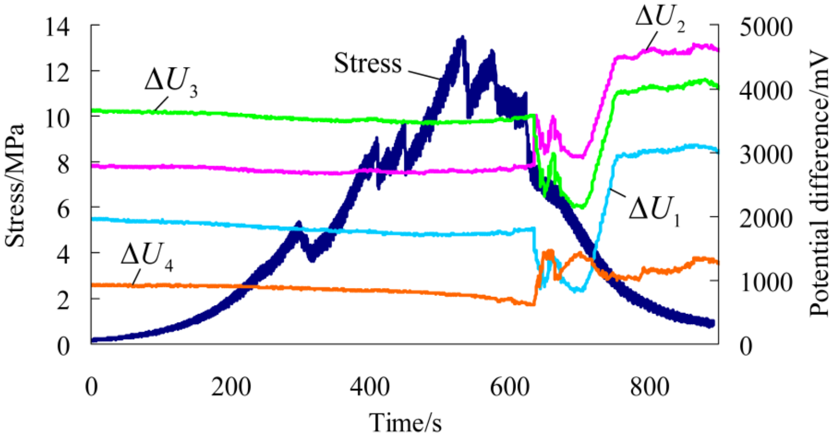


(**b**)

**Figure S2.** Stress-time curve and time-varying curve of the potential difference in each direction during the whole process of uniaxial compression for rock samples normal to the bedding direction. (**a**) Rock sample 6-2; (**b**) Rock sample 6-3.
